# State of the art: glioma-associated epilepsy—bridging tumor biology and epileptogenesis

**DOI:** 10.1186/s42466-025-00434-8

**Published:** 2025-10-10

**Authors:** Iris Divé, Anna-Luisa Luger, Dorothea Muench, Katharina J. Weber, Joachim P. Steinbach, Felix Rosenow, Frank Winkler, Pia S. Zeiner

**Affiliations:** 1 Dr. Senckenberg Institute of Neurooncology, University Medicine Frankfurt, Goethe University Frankfurt, Frankfurt, Germany; 2 Institute of Neurology (Edinger-Institute), University Medicine Frankfurt, Goethe University Frankfurt, Frankfurt, Germany; 3 Frankfurt Cancer Institute (FCI), Goethe University Frankfurt, Frankfurt, Germany; 4 University Cancer Center (UCT), University Medicine Frankfurt, Goethe University Frankfurt, Frankfurt, Germany; 5 German Cancer Consortium (DKTK), Partner Site Frankfurt/Mainz, DKFZ and University Hospital Frankfurt, Frankfurt, Germany; 6 Department of Neurology, Epilepsy Center Frankfurt Rhein-Main, University Medicine Frankfurt, Goethe University Frankfurt, Frankfurt, Germany; 7 Clinical Cooperation Unit Neurooncology, Neurology Clinic and National Center for Tumor Diseases, University Hospital Heidelberg, Heidelberg, Germany; 8 Department of Neurology, University Medicine Frankfurt, Goethe University Frankfurt, Frankfurt, Germany

**Keywords:** Glioma-associated epilepsy, Molecular classification, Tumor biology, Epileptogenesis, Cancer neuroscience

## Abstract

**Background:**

Glioma-associated epilepsy (GAE) is a frequent and clinically significant complication in neuro-oncological practice. Its prevalence varies across glioma subtypes and is influenced by tumor biology, cortical involvement, tumor size, extent of resection, and disease progression. Despite its substantial impact on quality of life and clinical outcomes, GAE remains underrepresented in neurological and neuro-oncological guidelines. Moreover, novel findings in molecular subtyping and their relevance to tumor biology and GAE pathogenesis are not yet adequately reflected in clinical frameworks. Here, we aim to provide a comprehensive synthesis of epidemiology, pathophysiology, and management strategies for GAE based on the recent advances in glioma biology, cancer neuroscience, and epileptology.

**Main body:**

This review highlights recent insights into the epidemiology, clinical impact, pathophysiology, and therapeutic strategies for GAE. We focus on both lower-grade gliomas, in which GAE is most prevalent over lifetime—particularly in tumors harboring isocitrate dehydrogenase (IDH) mutations—as well as high-grade gliomas where GAE remains a clinically relevant and complex issue. In addition to diffuse glioma subtypes, this review also addresses low-grade epilepsy-associated tumors (LEAT), a distinct and heterogeneous group with an inherently high risk of seizures. The pathomechanisms of GAE are reviewed with regard to glioma subtype-specific alterations of the tumor metabolism, neuroinflammation, increased glutamatergic activity, as well as the interaction between tumor cells and non-neoplastic cells. Key pathways implicated in both GAE and tumor biology include the IDH and mTOR signaling and a range of tumor related somatic mutations. With regard to the prognostic and therapeutic significance of GAE, we highlight the essential importance of accurate molecular tumor classification. In addition to reviewing common and tumor-specific side effects of anti-seizure medication (ASM), the emerging role of therapeutic approaches targeting both tumor growth and epileptogenesis is discussed.

**Conclusion:**

Glioma (subtype) specific mechanisms of epileptogenesis and selection of ASM is an emerging topic with future potential to improve the therapy of GAE and tumor growth alike.

## Background

Glioma-associated epilepsy (GAE) is a common and clinically significant complication in patients with gliomas [1, 2, 3]. Seizures affect not only the disease trajectory, but also the end-of-life periods [4]. Despite its high prevalence, GAE remains insufficiently understood and underrepresented in neuro-oncological treatment guidelines, especially in the context of distinct molecular glioma subtypes. Control of seizures is jeopardized by tumor progression, pharmacoresistance, and side effects of antiseizure medication (ASM). These often-complex clinical situations require personalized treatment strategies that integrate both neuro-oncological and epileptological considerations. Recent advances in molecular neuropathology [5] have improved our understanding of glioma heterogeneity. In addition, the emerging field of cancer neuroscience bears the potential to give rise to disease modifying drugs which simultaneously suppress seizure activity; at the same time, it can guide glioma (subtype) specific selection of ASM. However, the link between defined molecular glioma entities, their underlying tumor biology, and mechanisms of epileptogenesis have yet to be systematically explored. This review addresses this gap, aiming to provide a comprehensive synthesis of epidemiology, pathophysiology, and management strategies for GAE based on the current knowledge and recent advances in glioma biology and epileptology.

A key focus is placed on distinct pathophysiological features of molecular glioma subtypes, including alterations driven by isocitrate dehydrogenase (IDH) mutations, dysregulation of the mammalian target of rapamycin (mTOR) pathway, glutamatergic imbalance and the emerging field of cancer neuroscience. Low-grade epilepsy-associated tumors (LEATs) are also addressed as a separate group of low-grade neuroepithelial neoplasms with inherently high epileptogenicity.

By integrating recent advances in glioma biology and epilepsy research, we frame GAE within a translational neuro-oncological and epileptological perspective, highlighting the necessity for therapeutic strategies that, ideally, target both subtype-specific glioma biology and seizure pathophysiology. Therefore, we outline current and emerging ASM strategies, including targeted approaches linked to molecular glioma profiles and novel strategies investigated in clinical trials.

## Main text

### Understanding the prognostic impact of glioma-associated epilepsy in the molecular era

GAE is defined as the occurrence of one or more unprovoked seizures directly attributable to glioma diagnosis or progression, with the epileptogenic zone anatomically and temporally linked to the tumor. The diagnosis of GAE is based on clinical presentation supported by neuroimaging, EEG and exclusion of alternative seizure causes [6]. Besides the molecular and histological glioma type, the most relevant risk factors for GAE are the tumor location (especially cortical involvement) and the extent of resection. Additional factors include the presence of preoperative seizures and molecular alterations affecting glutamatergic signaling and neuronal excitability [7, 8, 9].

GAE develops in most patients with low-grade gliomas (LGG) over the course of the disease and also affects approximately half of patients with high-grade gliomas (HGG), particularly when adjusted for disease duration. LGGs have a high seizure prevalence ranging from 60–90%, depending on the LGG subtype. Seizures are often the first symptom in LGGs and remain a dominant clinical issue throughout the disease course [10]. Oligodendrogliomas exhibit the highest prevalence (65–72%), followed by low-grade astrocytomas (60–68%) [1, 2]. In HGGs, GAE occurs less frequently but remains clinically relevant, with a prevalence of 30–60% [1, 2]. Although prevalence at initial diagnosis is lower in HGG, it increases with disease duration, particularly in patients with prolonged survival [11, 12]. In IDH wildtype (wt) glioblastoma (GB), recurrence or worsening of seizures is often associated with tumor relapse, hence management of GAE in GB remains especially challenging [13].

From a neuro-oncological perspective, it is important to note that many earlier studies on GAE, conducted before the 2021 update of the WHO classification [5], did not distinguish HGGs according to IDH mutation status or other molecular markers. Hence, IDHwt GB were often analyzed along with IDH mutant (mt) gliomas. This represents a significant confounder in the assessment of HGG-related epilepsy, since IDHmt gliomas—including the higher WHO grades 3 and 4—show substantially higher seizure rates than IDHwt GB (WHO grade 4) [14, 15]. A major confounder to be addressed in future studies is the longer disease course of IDH-mt gliomas, which likely augments epileptogenicity but might not fully explain the difference to IDHwt gliomas. Tumor-intrinsic factors, such as the epileptogenic potential of the IDH mutation and other biological features distinguishing these entities, require better understanding in this context. It is necessary to specifically address the pathophysiological role of the IDH mutation in the context of GAE, considering the distinct biological and clinical behavior of IDHmt gliomas. Beyond IDH status, molecular stratification within IDHwt gliomas according to the current WHO classification demonstrates that histologically lower-grade gliomas (WHO grade 2 and 3) with a molecular GB-like profile (molecular GB IDHwt) exhibit a significantly higher incidence of GAE before diagnosis and a longer interval between seizure onset and tumor detection compared to classical IDHwt GB, suggesting a distinct clinical trajectory [16].

The prognostic impact of GAE is complex and context-dependent. Seizures both at diagnosis and in the later course of disease are a favorable prognostic marker in certain glioma subtypes, while refractory GAE and status epilepticus (SE) are negative prognostic indicators (Table 1). As such, seizures at presentation have been associated with prolonged OS in mixed cohorts of LGGs and HGGs [15, 17, 18], as well as in a recent study of molecularly stratified IDHwt GB [19]. In contrast, in a large recent study of over 500 IDHwt GB patients, no significant association was found with OS. Still, this study suggests a positive association of seizures and survival, both at diagnosis and later on, in the total mixed HGG cohort [18]. These data should be interpreted with caution and validated in strictly molecularly defined, non-mixed glioma cohorts that account for disease duration. This is particularly important when assessing the prognostic role of seizures, as it remains unclear whether disease duration is the primary driver of epileptogenicity—especially in LGGs—or whether it acts instead as an additional modifier alongside intrinsic tumor biology.

**Table 1:** Overview of molecular features, seizure prevalence, tumor biology, and implications for GAE across major glioma subtypes and LEATs. Tumor subtypes are categorized according to the 2021 WHO classification. Seizure prevalence is based on published literature and reflects variability. Abbreviations: IDH = isocitrate dehydrogenase; wt = wildtype; mt = mutant; GB = glioblastoma; LEAT = low-grade epilepsy-associated tumor; SEGA = subependymal giant cell astrocytoma; GAE = glioma-associated epilepsy

Glioma subtype	Molecular features	Seizure prevalence	Mechanistic hallmarks	Prognostic relevance of GAE
IDHmt gliomas	IDHmt	High	2-HG, glutamatergic dysregulation, network remodeling, cortical spread	GAE favorable; SE negative; seizure control with IDH inhibitors
GB	IDHwt	Moderate	neurotransmitter imbalances, neuron-to-glioma synapses	Prognostic role unclear (possibly favorable); SE negative predictor; cancer neuroscience increasingly relevant
LEAT	Several alterations incl. BRAFmt and mTOR-related pathways	Very high	intrinsic epileptogenicity, focal lesions, mTOR activation	Excellent tumor prognosis; epilepsy surgery crucial for seizure freedom
SEGA	TSC1/2mt	High	mTOR activation, synaptic reorganization	mTOR inhibitors: tumor control & seizure reduction

Importantly, refractory epilepsy and the occurrence of SE are linked to significantly worse quality of life and poor OS in GB patients, but also mixed cohorts with different tumor types independent of the tumor entity [20, 21]. Postoperative or new-onset epilepsy after initial treatment may indicate tumor progression and is also linked to worse outcomes [20, 22]. In sum, interpreting the prognostic relevance of GAE is closely tied to the molecular stratification of study cohorts. Many earlier studies allow for the hypotheses that seizures reflect a per se more favorable tumor biology, such as slower growth, cortical location, or molecular features like the IDH mutation or the 1p/19q codeletion in oligodendrogliomas, all parameters independently of GAE associated with a better prognosis. However, studies on seizure impact in mixed LGG/HGG cohorts without current molecular classification have limited validity. The prognostic significance of GAE in defined molecular glioma subtypes remains to be explored in detail. Future studies using state-of-the-art tumor diagnostics and controlling for confounders such as extent of resection, tumor location, and tumor size may help to disentangle these factors. This could move beyond attributing the association between GAE and better outcomes merely to earlier diagnosis or treatment bias, and instead offer insights into possible intrinsic biological mechanisms linking GAE to prognosis.

### Molecular and pathophysiological mechanisms of glioma-associated epilepsy

#### Glioma-associated epilepsy and IDH mutations

**Fig. 1:**
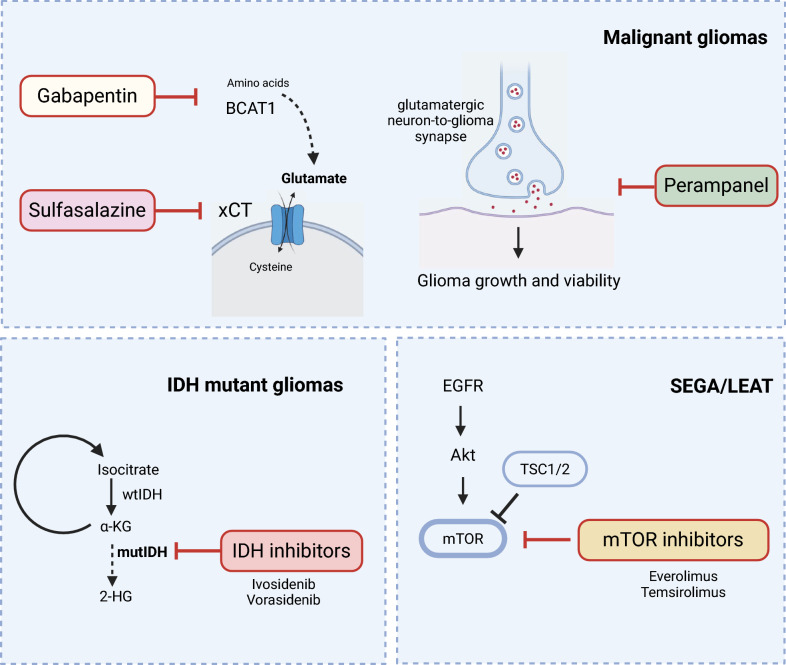
Emerging targeted concepts in the treatment of GAE. The figure provides an overview of targeted concepts that are currently being evaluated for the treatment of GAE. Abbreviations: 2-HG = 2-hydroxyglutarate; α-KG = α-ketoglutarate; BCAT-1 = Branched-chain amino acid transaminase 1; mTOR = mammalian target of rapamycin; IDH = isocitrate dehydrogenase; wt = wildtype; mt = mutant; LEAT = low-grade epilepsy-associated tumor; SEGA = subependymal giant cell astrocytomas. Created in https://BioRender.com

The pathophysiology of epileptogenesis in gliomas harboring IDH mutations has been well studied. Of the three isoforms, IDH mutations most frequently occur in IDH1. In the Krebs cycle, IDH1 catalyzes the oxidative decarboxylation of isocitrate to alpha-ketoglutarate (α-KG, also known as 2-oxoglutarate (2-OG)). In contrast, mutations in IDH1 lead to the production of D-2-hydroxyglutarate (2-HG). This is not only due to the decreased affinity of the enzyme for isocitrate, but also to a neomorphic enzymatic activity which reduces α-KG to 2-HG [23] (Fig. 1). 2-HG has been widely accepted as an oncometabolite with broad biological, immunological [24], and molecular effects [25, 26] not only in glioma cells, but also in the tumor microenvironment. Metabolically, 2-HG leads to the accumulation of reactive oxygen species, thereby causing oxidative stress and inducing autophagy [27, 28]. At the same time, 2-HG inhibits the branched-chain amino acid transaminase 1/2 (BCAT1/2), which impairs the generation of glutathione and glutamate. As a consequence, IDHmt tumors are highly dependent on glutaminolysis [28]**.**

While low glutamate levels appear to contrast with the high rate of epilepsy in IDHmt gliomas, the hyperexcitability arises from the structural similarity of 2-HG to glutamate. Indeed, the addition of exogenous 2-HG to rat cortical neurons increased their electrical activity, an effect that was countered by the NMDA antagonist AP5 [14]. Additionally, it has been proposed that 2-HG induces neuronal hyperexcitability via activation of the mTOR signaling pathway [29]. mTOR activation was accompanied by enhanced mitochondrial respiration and glycolytic activity. This was observed not only in glioma cells, but also in surrounding cells [24]. The exact mechanism by which mTOR signaling contributes to epileptogenesis is yet to be elucidated (see “Glioma-associated epilepsy and mTOR pathway alterations”).

#### Glioma-associated epilepsy and mTOR pathway alterations

From a neuro-oncological perspective, LEATs and subependymal giant cell astrocytomas (SEGA) represent the best-known examples of pathological mTOR activation. In these tumors, enhanced mTOR signaling contributes to both tumor growth and neuronal hyperexcitability [30, 32]. However, overactivation of the mTOR signaling pathway also appears to contribute to the epileptogenesis of malignant gliomas. In genetic mouse models of these tumors, specific mutations in phosphoinositide 3-kinase (PIK3)—which is located upstream of mTOR—lead to distinct variants of its catalytic subunit PIK3CA. These were related to particularly high brain hyperexcitability and reorganization of synaptic structure [33]. Taken together, these findings suggest that the PIK3-mTOR cascade plays a larger role in glioma biology and epileptogenesis than previously recognized.

The underlying molecular mechanisms of how mTOR signaling impacts hyperexcitability remain poorly understood. At the mechanistic level, tumor cell-intrinsic epileptogenesis involves mTOR-driven proliferation and alterations in cell morphology, including somatic hypertrophy and ectopic axon growth. mTOR activation also causes changes in synaptic structure and protein expression that increase neuronal excitability [32]. Given its role in cell proliferation, growth and morphology [34], mutations affecting the mTOR pathway are also likely to contribute to the tumor-driven circuit remodeling that has been observed in recent studies.

Clinically, SEGAs in the context of tuberous sclerosis complex respond to mTOR inhibition resulting in significant tumor shrinkage and seizure reduction [35]. Emerging evidence suggests similar mechanisms may also be active in certain LEATs, especially those with DYRK1A mutations. A recent analysis of 1.386 epilepsy-associated lesions revealed novel molecular associations, including DYRK1A and EGFR mutations, in epileptogenic tumors. Notably, mTOR pathway activation was consistently observed in all DYRK1A-mutant cases, suggesting that DYRK1A alterations may represent both diagnostic markers and potential therapeutic targets [36]. Hence, integrating molecular profiling—particularly assessment of DYRK1A, EGFR mutations and mTOR activation—could inform personalized treatment strategies.

#### Epileptogenesis in low-grade epilepsy-associated tumors

As a special category, low-grade epilepsy-associated tumors (LEATs) comprise a heterogeneous group of predominantly low-grade, often glioneuronal neoplasms that typically present with chronic, drug-resistant epilepsy in children and young adults [37]. Gangliogliomas (GG) and dysembryoplastic neuroepithelial tumors (DNETs), both classified as glioneuronal tumors, account for the majority of LEATs besides additional rare entities [38, 39]. Due to the histopathological diversity of LEATs and the limited number of cases, there is considerable interrater variability, making molecular and genetic testing essential for accurate diagnosis [39]. Somatic mutations are relevant for LEAT, especially GG [40]. LEATs have the highest seizure rates among brain tumors, with GGs and DNETs comprising seizure rates above 80% or even 90% [1, 2]. Diffuse LGGs and HGGs are not traditionally classified as LEATs, though they can be highly epileptogenic, and some consider certain astrocytomas and oligodendrogliomas to fall within the LEAT spectrum [37, 40].

LEATs are the second most common indication for epilepsy surgery after hippocampal sclerosis [41]. Epileptogenesis in LEATs is believed to be a multifactorial process although its mechanisms are not yet fully understood [39, 40]. BRAF^V600E^ mutations may lead to epileptogenicity in developing neurons, through an increased expression of RE1-silencing transcription factor (REST) [42]. Besides the better explored BRAF^V600E^ mutations, other alterations, such as of PTPN11/KRAS/NF1 or the mTOR pathway, were found in LEATs. Pointing at similar disturbances in cellular pathways between neoplastic and non-neoplastic epileptogenesis, the latter alterations have already been characterized and investigated in other epilepsy disorders like malformation of cortical development [36, 43, 44]. Moreover, in mouse models of GG, it has been shown that factors released by GG cells have a direct effect on neurons and could potentiate neuronal network synchronicity [45]. As stated above, the work by Boßelmann et al. recently established a link between LEATs and alterations in DYRK1A- and EGFR-related pathways.

#### Glutamatergic dysregulation in high-grade gliomas: linking epileptogenesis to glioma tumor biology

In HGGs, increased extracellular glutamate concentrations are considered a key contributor to epileptogenesis. Glioma cells have been shown to induce regional brain hyperexcitability by releasing high levels of glutamate [46], possibly due to increased expression of the glutamate antiporter xCT [47]. At the same time, glutamate reuptake, primarily mediated by astrocytes, is impaired. This is a result of widespread astrocytic dysfunction and altered expression of transmembrane transporters, such as the excitatory amino acid transporter 2 (EAAT2). Increased extracellular glutamate concentrations contribute to the occurrence of seizures via increased signaling activity of NMDA receptors which stimulates the influx of calcium, and the diminishment of GABA receptors [48].

Within the glioma microenvironment, however, the effects of increased glutamate levels go far beyond epileptogenesis, as evidenced by a series of recent publications. Glutamate has been shown to promote glioma cell growth, viability and invasiveness [49]. At the ultrastructural level, this was attributed to glutamatergic neuron-to-glioma synapses [50] which involve cellular processes of glioma cells termed tumor microtubes (TM) [51, 52]. Gutamatergic neuron-to-glioma synapses of the AMPAR type enable glioma cells to integrate into the peritumoral neuronal network and function as a connection between glioma cells and neurons. Neuronal hyperexcitability activates glioma cells and is associated with glioma growth and worse survival, including in glioblastoma patients [52, 53, 54]. The growth-enhancing effects are based on glutamate signal transduction as well as on paracrine mechanisms such as the secretion of the synaptic protein neuroligin-3 (NLGN3) [54] and the brain-derived neurotrophic factor (BDNF) [55]. In addition, neuron-to-glioma synapses of the AMPAR type are even located on invasive glioblastoma cells and drive brain colonization [56]. Interestingly, in H3K27M-altered diffuse midline glioma, tumor cell proliferation occurs via GABA_A_ receptors which are *excitatory*, resembling neurodevelopmentally early ones and, paradoxically, can thus be potentiated by benzodiazepines like lorazepam [57], which suggests subtype-specific mechanisms of neuron-to-glioma synapses. Notably, the complex interaction between glioma cells and neurons appears to be mediated not only by neurotransmitters but also by aberrant ion flux, causing spreading depolarization [58]. Whether ion dysregulation arises as a consequence of neuronal hyperexcitability or constitutes an initiating event that precipitates neuronal dysfunction remains to be elucidated.

The clinical relevance of the neurogliomal interaction is highlighted by the observation that the remodelling of neuronal circuits through NGS is associated with shorter overall survival in GB [53, 59]. In line with this, neuron-to-glioma synapses have not been observed in more benign tumor types such as oligodendrogliomas (in contrast to grade 2 to 4 astrocytomas) or meningioma [52]. In sum, the emerging field of cancer neurosciences links network hyperexcitability to tumor biology and offers a molecular explanation for the co-occurrence of epileptic seizures and tumor progression. At the same time, the results of cited studies provide a rationale for (pre-)clinical studies with so-called disease-modifying ASM in the context of GAE (see below: Section “Targeted and emerging concepts”).

#### The role of neuroinflammation in GAE

Neuroinflammation is a key driver of GAE, with multiple mechanisms linking inflammatory processes to neuronal hyperexcitability and seizure generation [60]. The glioma microenvironment is characterized by activated microglia and macrophages, astrocytes with inflammatory functions, disruption of the blood–brain barrier (BBB), and release of pro-inflammatory cytokines that directly promote brain tumor-induced neuronal hyperexcitability. For example, IL-1β and TNF-α enhance excitatory synaptic transmission and suppress GABAergic signaling, thereby lowering the seizure threshold [60, 61, 62]. TNF-α additionally upregulates neuronal voltage-gated Na⁺ channels, further fostering hyperexcitability [62]. Moreover, the inflammatory milieu amplifies glutamatergic signaling, which is both epileptogenic and protumorigenic.

There is a bidirectional relationship between seizures and neuroinflammation: seizures induce neuroinflammatory responses, while inflammation in turn promotes seizure activity [63]. Seizure activity activates microglia via the P2X7 receptor, proposed as a gatekeeper of neuroinflammation [64]. Astrocytes also play a pivotal role by releasing cytokines and chemokines,in SE astrocytes are activated through interactions with neurons, microglia, and BBB dysfunction [65]. Seizures and pro-inflammatory mediators can disrupt the BBB, facilitating infiltration of immune cells and soluble mediators into epileptogenic tissue [66, 67]. In addition to CNS-resident cells, peripheral immune cells with pro-inflammatory and cytotoxic functions infiltrate epileptogenic glioma lesions [68].

Neuroinflammation also intersects with other pathogenic pathways in GAE. For example, EGFR mutations correlate with increased immune cell infiltration [69]. Pro-inflammatory cytokines such as IL-1β and TNF-α promote excessive glutamate release through regulation of glutaminase activity [70], thereby linking neuroinflammation with cancer neuroscience. Further, in ganglioglioma models, tumor-derived factors have been shown to directly modulate neuronal activity and enhance network synchronicity [45, 71]. Taken together, neuroinflammation is a major contributor to GAE with extensive literature available, but its in-depth discussion is beyond the scope of this review.

### Current and emerging strategies for antiseizure medication

#### General considerations and challenges

In the 2005 definition of the International League Against Epilepsy (ILAE), the diagnosis of epilepsy is given after one unprovoked seizure when the recurrence risk is > 60% over the next 10 years. Since seizure recurrence risk is high in brain tumor patients, consensus recommends starting ASM after the first seizure [72]. Prophylactic and/or perioperative prescription of ASM is not recommended for patients with newly diagnosed brain tumors who have not experienced seizures [73].

The potential of tumor therapy to reduce seizure risk has been investigated in several studies. Surgical resection plays a key role in seizure reduction, most prominently in LEATs, in which early and complete removal of the epileptic zone (i.e. the tumor with a surrounding tissue margin) is the most effective method for seizure control [74]. Resection also contributes to seizure reduction in IDHmt glioma, particularly if extent of resection of > 80% is achieved [75]. Similar effects are observed for GB [76]. With regard to adjuvant treatment, both radiotherapy and chemotherapy have been shown to reduce seizure frequency in patients with IDH-mt gliomas [12, 77, 78]. Commonly used anti-edematous agents, such as bevacizumab, may also contribute to seizure control in GB patients [79].

Despite the antiseizure effect of tumor treatment, a substantial proportion of patients with diffuse glioma continue to experience seizures or SE [80]. This highlights that, in most cases, tumor treatment alone cannot replace pharmacological seizure control. Studies on ASM efficacy and safety in GAE are predominantly retrospective, which limits evidence-based decision-making [81]. Furthermore, findings on the selection and popularity of certain ASMs can be confounded by presumed antitumor effects, such as the once-suggested but now contested survival benefits of valproic acid and levetiracetam in glioma patients [82]. While guidelines for the treatment of (focal) epilepsy may inform GAE management, the distinct pathomechanisms of epileptogenesis and the specific clinical challenges of GAE highlight the urgent need for prospective, disease-focused studies.

#### Pharmacological treatment

In the context of GAE, there are certain requirements for ASM: These include, of course, effective seizure suppression and good tolerability, but also rapid onset of action, low risk of sedation and encephalopathy and little to no interaction with the antitumor treatment. As stated above, prospective, randomized trials comparing the safety and effectiveness of ASM classes for GAE are sparse (Table 2). This is reflected in the fact that, out of 66 articles included in a systematic review, only two were randomized controlled trials [81]. De Bruin and colleagues analyzed the efficacy of seizure control and dropout rates based on a weighted average, which takes into account the number of patients enrolled in a given study. The authors found the highest rate of seizure freedom at 6 months for phenytoin. After twelve months, pregabalin and levetiracetam were the most effective. For seizure reduction of ≥ 50%, levetiracetam had the best outcomes at both six and twelve months. Both phenytoin (34%) and pregabalin (41%) showed high treatment failure rates (defined as discontinuation of the ASM or the addition of a second substance) at 12 months. Similar results were observed in another recent retrospective study [83]. When compared to enzyme-inducing ASM (carbamazepine, oxcarbazepine, phenytoin, phenobarbital, levetiracetam had a lower risk of treatment failure due to adverse events, while the failure rate due to uncontrolled seizures was similar. Also, enzyme-inducing ASM can unfavourably impact the metabolization of chemotherapeutic drugs as well as small-molecule or kinase inhibitors [84, 85]. First-generation drugs such as phenobarbital and phenytoin, but also more recently approved agents like cenobamate, are usually administered with caution because they interact with a broad range of drug classes, including sleep medications and antidepressants, which are frequently prescribed to brain tumor patients. Overall, levetiracetam stands out as the ASM for which the existing literature provides the most substantial body of evidence. These results are mirrored by a survey of the European Association of Neurooncology (EANO) [72], in which the ASMs most frequently used for the treatment of GAE were second-generation substances, particularly levetiracetam and lacosamide. To date, only one prospective study exists on lacosamide in monotherapy [86]. The study reported a rate of seizure freedom of 55% at 6 months with good tolerability (drop-out rate of 1.5%). Although lamotrigine was shown to be similarly effective as lacosamide, its use is generally less favoured due to the need of dose titration [87]. However, for young patients with good oncological prognosis such as LEAT patients, lamotrigine may be a good choice because it has the least amount of neurocognitive side effects. While valproic acid may be considered less suitable given the risk of coagulopathy [63], encephalopathy [88] and the inhibition of the CYP enzyme class, it is one of the most used ASM in the Netherlands and non-European countries. In a retrospective analysis of levetiracetam versus valproic acid in first-line treatment in glioma patients with GAE, the substances did not differ in their toxicity. However, levetiracetam showed better seizure control [89].

**Table 2:** Overview of ASM monotherapy for GAE with evidence from clinical studies. The table summarizes the current evidence for the indicated substances from clinical studies. Abbreviations: RCT = randomized-controlled trial

Substance	Evidence	Cave
Carbamazepine	Retrospective, prospective	Modulation of CYP enzymes
Lacosamide	Retrospective	QTc interval
Lamotrigine	Retrospective	Latency due to dose titration, allergic reactions, interactions
Levetiracetam	Retrospective, prospective, RCT	Psychiatric side effects, dose reduction in patients with impaired GFR
Oxcarbazepine	Prospective	Modulation of CYP enzymes, hyponatremia
Phenobarbital	Retrospective	Drug interactions
Phenytoin	Retrospective, RCT	Drug interactions
Pregabalin	RCT	Dizziness, withdrawal symptoms
Topiramate	Retrospective, prospective	Cognitive side effects, teratogenicity
Valproic acid	Retrospective	Drug interactions, coagulopathy, teratogenicity

About 30% of brain tumor patients need duo- or polytherapy for seizure control. The evidence supporting combination therapies remains limited, primarily due to the heterogeneity of the studies, most of which are retrospective. Furthermore, several studies fail to provide detailed information on the specific drug combinations investigated. The highest seizure freedom rates were reported for the combination of ASM with either phenytoin or topiramate [81]. Add-on perampanel showed good results for ≥ 50% seizure reduction at 6 and 12 months. These findings contrast with the common practice among EANO members, who most frequently turned to lacosamide, lamotrigine and valproic acid after treatment failure had occurred [72]. The efficacy of add-on lacosamide [90] or lamotrigine [87] was reported from retrospective studies. The combination of levetiracetam and valproic acid had better seizure control compared to other duotherapy regimens in a retrospective analysis of 355 patients [91], and no differences in the rate of adverse effects were observed.

Taken together, the current literature underscores the value of levetiracetam in GAE monotherapy. However, this contrasts the SANAD II study comparing monotherapy of levetiracetam or zonisamide to lamotrigine in individuals with newly diagnosed focal epilepsy and found lamotrigine to be more effective and better tolerated than levetiracetam [92]. This finding once again underscores the dilemma of selecting an appropriate ASM for GAE in the absence of dedicated studies. Caution should be taken in patients with a history of psychiatric disorders. Although brivaracetam has been reported to less frequently induce behavioural changes [93], it has not been evaluated as monotherapy specifically for GAE. While valproic acid was proven to efficiently reduce seizure frequency in GAE, it seems less preferable to due interaction with other drugs, weight gain, teratogenicity and the risk of coagulopathy.

When implementing combination therapy, it is advisable to select agents with differing mechanisms of action, in alignment with established treatment strategies for primary epilepsy syndromes. The data for combination therapy are limited. The best formal evidence for combination therapy is available for the combination of levetiracetam with valproic acid. However, based on the considerations elaborated above and clinical experience in neuro-oncology, add-on therapy with lacosamide is preferred in many centers. Add-on lamotrigine or perampanel may be alternative options.

#### Targeted and emerging concepts

The molecular classification of brain tumors and the growing biological understanding of epileptogenesis in distinct glioma entities have set the stage for the concept of targeted ASM that could not only reduce seizure frequency but also positively influence the course of tumor disease (Fig. 1).

Solid evidence exists for mTOR inhibitors, such as everolimus, which have demonstrated consistent efficacy in the treatment of SEGAs in patients with tuberous sclerosis complex, supported by early phase II data [94], the pivotal phase III EXIST-1 trial [95], and phase III evidence from the EXIST-3 study demonstrating substantial seizure reduction [96]. Preclinical glioma models using mTORC1/2 inhibitors (e.g. AZD8055) have shown seizure suppression, highlighting direct modulation of epileptogenic circuits beyond tumor size reduction [31]. These findings align with the molecular signature of mTOR hyperactivation in DYRK1A-mutant lesions and support expanded use of targeted therapies in defined molecular subgroups [36].

In LEATs, multiple strategies for the development of novel therapeutic approaches are under investigation. The presence of BRAF^V600E^ mutations in GG could enable targeted treatment strategies with BRAF inhibitors [44].

In IDHmt gliomas, IDH inhibitors were recently shown to prolong progression-free survival and have therefore emerged as a novel treatment option [97]. Mechanistically, they reduce intratumoral 2-HG concentrations [98]. IDH inhibitors have been reported to reduce seizure frequency in single case reports [99], and also in a recently updated analysis of the INDIGO trial presented at the SNO annual meeting in 2024.

Resulting from the field of cancer neurosciences, the antiseizure effects of several substances involved in the neuron-to-glioma synapses are currently under investigation. An example hereof is perampanel, an AMPA receptor antagonist that inhibits malignant neuron-to-glioma synapses in vivo and thus reduces glioblastoma growth [52] and invasion [56]. The potential of perampanel to modulate the neuron-glioma synaptic network and reduce seizure frequency will be investigated in the PerSurge phase II trial [100]. Meclofenamate, which impairs the formation of neuron-to-glioma synapses and in vivo inhibits pharmacologically induced seizure activity through ion channel inhibition [101], is currently under clinical investigation in the MecMeth/NOA-24 trial [102]. In a retrospective study, administration of gabapentin following surgical resection of newly diagnosed GB correlated with improved patient survival [103]. The effect of gabapentin in combination with sulfasalazine, memantine and standard-of-care radiochemotherapy is currently investigated for GB patients in the GLUGLIO phase Ib/II trial [104].

## Conclusions

GAE is a frequent and clinically significant challenge in neuro-oncology and epileptology, shaped by the molecular subtype, localization, and biology of the underlying glioma. While GAE may indicate favorable tumor features, such as slower-growing, cortically located gliomas with IDH mutations, its exact prognostic relevance in precisely stratified cohorts has yet to be determined. Integrating molecular glioma classification with mechanisms of epileptogenesis (including particular mechanisms involved in GAE such as IDH and mTOR pathway alterations, glutamatergic dysregulation, and cancer neuroscience) is crucial for improving diagnosis, prognostication, and treatment. Personalized strategies that target both tumor growth and seizure activity, while accounting for pharmacoresistance and glioma-specific biology, are essential for advancing patient care.

## Data Availability

Not applicable.

## Abbreviations

2-HG: 2-Hydroxyglutarate
α-KG: α-Ketoglutarate
ASM: Antiseizure medication
BBB: Blood–brain barrier
BCAT-1: Branched-chain amino acid transaminase 1
DNET: Dysembryoplastic neuroepithelial tumor
EGFR: Epidermal growth factor receptor
GB: Glioblastoma
GAE: Glioma-associated epilepsy
IDH: Isocitrate dehydrogenase
LEAT: Low-grade epilepsy-associated tumor
LGG: Lower-grade glioma
mTOR: Mammalian Target of Rapamycin
mt: Mutant
OS: Overall survival
PIK3: Phosphoinositide 3-kinase
SE: Status epilepticus
SEGA: Subependymal giant cell astrocytoma
TSC1, TSC2: Tuberous sclerosis protein 1 and 2
wt: Wildtype
