# Publisher Correction: State of the art: glioma-associated epilepsy—bridging tumor biology and epileptogenesis

**DOI:** 10.1186/s42466-025-00455-3

**Published:** 2025-12-15

**Authors:** Iris Divé, Anna-Luisa Luger, Dorothea Muench, Katharina J. Weber, Joachim P. Steinbach, Felix Rosenow, Frank Winkler, Pia S. Zeiner

**Affiliations:** 1 Dr. Senckenberg Institute of Neurooncology, University Medicine Frankfurt, Goethe University Frankfurt, Frankfurt, Germany; 2 Institute of Neurology (Edinger-Institute), University Medicine Frankfurt, Goethe University Frankfurt, Frankfurt, Germany; 3 Frankfurt Cancer Institute (FCI), Goethe University Frankfurt, Frankfurt, Germany; 4 University Cancer Center (UCT), University Medicine Frankfurt, Goethe University Frankfurt, Frankfurt, Germany; 5 German Cancer Consortium (DKTK), Partner Site Frankfurt/Mainz, DKFZ and University Hospital Frankfurt, Frankfurt, Germany; 6 Department of Neurology, Epilepsy Center Frankfurt Rhein-Main, University Medicine Frankfurt, Goethe University Frankfurt, Frankfurt, Germany; 7 Clinical Cooperation Unit Neurooncology, Neurology Clinic and National Center for Tumor Diseases, University Hospital Heidelberg, Heidelberg, Germany; 8 Department of Neurology, University Medicine Frankfurt, Goethe University Frankfurt, Frankfurt, Germany

**Neurological Research and Practice (2025) 7:73**

10.1186/s42466-025-00434-8.

Due to a mistake on the publisher’s side during the incorporation of proof corrections, some of the references were not cited correctly within the text. This has now been amended and all references in the reference list were re-arranged to reflect this. The original article has been corrected.

